# Effects of Calf Starter Neutral Detergent Fiber Levels and Weaning on Growth and Rumen Microbial Diversity of Holstein Calves

**DOI:** 10.3390/ani16091316

**Published:** 2026-04-25

**Authors:** Özge Sayın Özdemir, Umair Ahsan, Ifrah Raza, Özcan Cengiz

**Affiliations:** 1 Department of Animal Nutrition and Nutritional Diseases, Faculty of Veterinary Medicine, Aydın Adnan Menderes University, Aydın 09016, Türkiye; raza_ifrah@hotmail.com (I.R.); ocengiz@adu.edu.tr (Ö.C.); 2 Department of Plant and Animal Production, Burdur Vocational School of Food, Agriculture and Livestock, Burdur Mehmet Akif Ersoy University, Burdur 15030, Türkiye; uahsan@mehmetakif.edu.tr; 3 Center for Agriculture, Livestock and Food Research, Burdur Mehmet Akif Ersoy University, Burdur 15030, Türkiye

**Keywords:** calves, genus, Holstein, microbial diversity, neutral detergent fiber, phylum, weaning

## Abstract

A study was conducted to evaluate the effect of neutral detergent fiber (NDF) levels of calf starter and weaning time on growth, rumen fermentation characteristics, serum metabolites, and rumen microbial diversity of Holstein calves. A total of 24 newly born male Holstein calves were randomly distributed to four groups in a completely randomized design with a 2 × 2 factorial arrangement of NDF levels (14% and 24%) and weaning time (d 44 and d 54). There was no interaction between starter NDF levels and weaning time for any trait except rumen acetic acid in the immediate post-weaning phase (*p* = 0.013). Starter NDF levels had no effect on growth, feed intake, and hay intake. Late-weaned calves had greater (*p* = 0.050) weight gain in the pre-weaning phase whereas, early-weaned calves showed greater weight gain (*p* = 0.004) and starter intake (*p* = 0.004) in the post-weaning phase although overall weight gain, and starter and hay intakes were not affected by weaning time. Rumen pH, ammonia nitrogen, and most volatile fatty acids remained unaffected by starter NDF levels and weaning except isobutyric acid which was greater in calves fed 24% NDF starter (*p* = 0.001) in the immediate post-weaning and isovaleric acid which was greater in early-weaned calves (*p* = 0.044) at the end of experiment. Serum metabolites were largely affected (*p* < 0.05) by starter NDF levels and weaning time in the pre-weaning phase only. Alpha diversity of rumen microbes was greater and chaotic in 14% NDF starter group (early- and late-weaned) in the pre-weaning phase which converged in the immediate post-weaning phase and diverged on starter NDF basis at the end of experiment. Microbial ecology at phylum and genus levels composition were greatly driven by starter NDF levels in the pre-weaning phase, by weaning time in the immediate post-weaning phase, and two distinct bifurcated microbial ecologies based on starter NDF levels appeared at the end of experiment. In conclusion, the comparable growth with distinct microbial diversity but largely in favor of 24% NDF starter suggests that calves can be subjected to early weaning with 24% starter NDF levels for smooth transition from liquid to solid feed in Holstein calves.

## Simple Summary

Feeding dairy calves early in life determines the future performance. We studied the response of calves to high-fiber feeds when they are forced to stop drinking milk earlier than usual. Initially, early withdrawal of milk and high-fiber feeds posed a challenge; however, they served towards the healthy development of calf’s digestive system. Microbes in the digestive system of calves appeared to adapt to the feeds. Low-fiber feeds helped develop the rapidly fermenters whereas, high-fiber feeds supported a highly specialized mix of microbes perfectly suited to degrade the tough plant cell walls. Interestingly, calves fed low- or high-fiber as well as early or late milk withdrawal had comparable growth by the end of the study (three months). It implies that farmers can use high-fiber natural feeds and wean the calves earlier than usual, supporting a healthy development of digestive system without negative consequences on the growth of calves.

## 1. Introduction

The first three months in the life of a calf is a critical period that lays the foundations for growth performance, immune development, and rumen functions. Feeding strategies adopted in this period have a carry-over effect on animal health as well as economic stability [1]. The improved immunity and welfare of calves are largely dependent on the implementation of an effective feeding program [2] that is critical in the second and third months of life to achieve these goals. In practice, calves are subjected to milk or milk replacer feeds that not only are labor-intensive but also pose a significant expense owing to the high cost of these inputs. An expedited transition of calves from liquid to solid feeds (early weaning) may reduce the milk feeding cost to a significant extent in addition to the labor requirement [3]. However, early weaning requires meeting the nutritional requirements for optimal growth and development since it influences calf health, growth efficiency, and long-term productivity. In addition, accelerated rumen development to enable quicker transition and to incentivize greater growth rate requires assistance in solid feed intake.

It has been reported that excessive milk intake delays solid feed intake in calves [4]. Therefore, early calf weaning requires limiting the milk allowance of calves to approximately 10% of birth weight while encouraging solid feed intake [5]. Early calf weaning shortens the milk consumption period and increases the solid feed intake, thus aiming to activate rumen functions earlier [6,7]. It allows maximization of roughage and concentrates (calf starter) intake in calves assisting in early weaning typically as early as 3 to 4 weeks after birth [8]. However, achieving a successful early weaning needs high-quality calf starter [6] and early access to clean water since water consumption is directly proportional to started feed intake and greater body weight gain [9].

As the calf transitions to a functional ruminant, the initiation of rumen microbial fermentation and the production of short-chain fatty acids (especially butyrate and propionate) are crucial for the development of the rumen epithelium [1,10]. These biochemical changes in the rumen, coupled with solid feed consumption, also shape ruminal morphology and the microbiome [11]. Early rumen development is a key to early calf weaning. Rumen development in early-weaned calves is directly influenced by the composition of calf starter. Structural carbohydrates, particularly fiber, promote the proliferation of rumen microorganisms and early colonization of fibrolytic populations [12,13] releasing acetate and butyrate volatile fatty acids (VFAs) that play an important role in the development of rumen papillae [6]. Calf starter including too-low fiber increases the risks of rumen acidosis that may limit the development of rumen muscle layer. Therefore, an appropriate amount of fiber in calf starter is critical for both a successful weaning, and long-term rumen health and calf performance [12]. Given that, it seems that early weaning and fiber content of solid feed may have complementary effects thereby resulting in earlier activation of rumen functions. Nonetheless, calf starter remains the sole nutritional source for calves during the early life implying that fiber content of calf starter is of critical importance. Accordingly, addressing these two factors together is crucial not only in reducing the milk consumption economically but also in shaping the rumen microbiota in calves in a timely and effective manner. Accordingly, we hypothesized that calf starter fiber levels might help early calf weaning by improving the growth and early establishment of rumen microbiota. Therefore, the present study was conducted to evaluate the effects of different fiber levels in calf starter and weaning time on growth, serum metabolites, rumen fermentation, rumen microbial diversity.

## 2. Materials and Methods

This study was conducted in Spring season of 2021 at a private dairy farm located in Aydın, Türkiye as the coordinates 37°48′03 N 28°01′10 E. All the methods and procedures involved in the study were in line and approved by the local animal care and use committee of Aydın Adnan Menderes University, Türkiye (protocol no. 2021/010).

### 2.1. Study Design

The study was carried out as a completely randomized design with 2 × 2 factorial arrangement of weaning time (d 44 and d 54) and neutral detergent fiber (NDF) levels in calf starter (14% and 24%) comprising 24 males Holstein calves randomly and equally assigned to 4 experimental groups. The study lasted for 90 days.

### 2.2. Animals

A total of 24 male Holstein healthy calves, with similar body weight, and born to cows at second or third calving, were procured from a large intensive commercial dairy farm (Atasancak Acıpayam Tarım İşletmesi Sanayi ve Ticaret A.Ş., Acıpayam, Denizli, Türkiye) and distributed randomly into four groups, each comprising 6 calves. All the calves were enrolled in the study simultaneously. Calves were housed in individual barns with slatted floor proving a 2.2 m^2^ covered area and a 1.42 m^2^ open area. This setting was continued until the end of the experiment.

### 2.3. Feeding

Each calf was fed a total of 4 L of Lactolac milk replacer (23% crude protein and 19% crude fat; Lacto Production, Bretagne, France) two times a day (0800 and 2000 h in the morning and evening, respectively) until weaning time (either 44 or 54 days according to the group). Milk replacer was prepared to provide 150 g/L milk replacer. Four days before weaning, the number of milk replacer feeding was reduced to 1 time a day. Calf starter diets were formulated to meet or exceed the nutrient requirements of calves according to the recommendations described by NRC [14]. Crude fiber levels were balanced according to the groups by adjusting the feedstuffs. Ad libitum access to calf starter was ensured to all the calves throughout the experiment. After weaning, calves were provided with ad libitum access to wheat hay. Composition of starter diets and wheat hay has been shown in Table 1.

### 2.4. Nutrient Composition of Diets and Feeds

Calf starter and hay were analyzed for dry matter, crude protein, ether extract, and crude ash according to the methods described by Association of Official Analytical Chemists [15]. Crude fiber, ash-free neutral detergent fiber after amylase treatment (aNDFom), and ash-free acid detergent fiber (ADFom) fractions were analyzed according to van Soest method [16] using ANKOM 200/220 fiber analyzer (ANKOM Technology, Macedon, NY, USA).

### 2.5. Growth and Feed Intake

Calves were individually weighed at 1200 h on a weekly basis to assess the live weight starting from the commencement until the end of experiment. Weight gain was computed using these live weight data. Milk replacer and calf starter intakes were monitored by recording the quantities offered and not consumed at the end of the day. Roughage consumption was monitored on daily basis after the weaning of calves. NDF intake in the pre-weaning and post-weaning periods and total NDF intake were calculated using the starter and hay intake data and their NDF contents. In addition, total NDF intake contributed by starter and hay were calculated.

### 2.6. Serum Metabolites

Blood samples (20 mL) were collected, within 2–4 h after feeding, from each calf 2 days before weaning (d 42 or d 52), 2 days after weaning (d 46 or d 56), and at the end of the experiment (d 90) into the vacutainers with gel activator by venipuncture technique of vena jugularis. The samples were allowed to clot followed by centrifugation at 4000 rpm for 10 min to separate the sera. Total protein (TP), glucose (GLU), blood urea nitrogen (BUN), cholesterol (CHOL), beta-hydroxybutyric acid (BHBA), glutamate dehydrogenase (GDH), and gamma-glutamyl transferase (GGT) were measured in serum using commercial reagent kits (Randox Laboratories Ltd., Crumlin, UK) and an automatic photometric plate reader (Randox Imola Biochemsitry Analyzer; Randox Laboratories Ltd., Crumlin, UK) according to manufacturer’s recommendations.

### 2.7. Rumen Fermentation Characteristics

Approximately 100 mL rumen fluid was collected from each calf 2 days before weaning (d 42 or d 52), 2 days after weaning (d 46 or d 56), and at the end of the experiment (d 90) using rumen catheter 4 h after morning feeding. pH was immediately measured using a pH meter followed by sieving the fluid through four layers of cheesecloth. Sulphuric acid (180 μL) was added to 9 mL of rumen fluid, subjected to centrifugation at 2000× *g* for 15 min, and 1.5 mL supernatant was separated into Eppendorf tubes to analyze the VFAs concentrations (Erwin et al., 1961) [17] using gas chromatography (7890B GC System, Agilent Technologies, Inc., Santa Clara, CA, USA) fitted with flame ionization detector. Following program was used to analyze VFAs: sample 1 μL, temperature 100–200 °C (10 °C/min for 10 min), injector temperature 250 °C, detector temperature 300 °C, nitrogen gas as carrier with flow speed of 1 mL/min, and capillary column of 30 m × 0.25 mm × 0.25 μm dimensions (DB-WAX GC column, Agilent Technologies, Inc., CA, USA). Acetic, propionic, butyric, isobutyric, valeric, and isovaleric acids were measured as VFAs against the standard (Sigma-Aldrich, St. Louis, MO, USA).

Besides measuring the VFAs, ammonia nitrogen (NH_3_-N) was measured in rumen fluid. For this purpose, 10 mL of sieved rumen fluid was mixed with 1 mL of 50% sulphuric acid. NH_3_-N was measured by a colorimetric method using NH_3_-N test kit (HI 93733-01, Hanna Instruments Inc., Woonsocket, RI, USA) and a photometer (HI 96733, Hanna Instruments Inc., RI, USA) according to manufacturer’s recommendations.

### 2.8. Rumen Microbial Diversity

Rumen microbiota analysis was conducted for quantitative assessment of overall bacterial composition of rumen of calves. To this end, 1 g rumen fluid mixed with 10 mL of physiological saline was homogenized in a tissue lyser at 4 °C for six cycles of 30 s each to settle coarse particles and associated bacteria. Supernatant was separated from each sample and subjected to DNA extraction using QIAamp PowerFecal Pro DNA kit (Qiagen Hilden, Germany) was used to extract DNA from the rumen fluid, followed by verification of concentration and purity of DNA using a NanoDrop spectrophotometer and Qubit fluorometer (Thermo Fisher Scientific, Waltham, MA, USA). The full-length 16S rRNA gene was amplified using the standard universal 16S primers 27F (5′-AGAGTTTGATCMTGGCTCAG-3′) and 1492R (5′-TACGGYTACCTTGTTACGACTT-3′) by following the PCR thermal cycling as initial denaturation at 95 °C for 5 min; 30 cycles of denaturation at 95 °C for 1 min, annealing at 55 °C for 1 min, extension at 72 °C for 90 s; followed by final extension at 72 °C for 7 min. For each PCR reaction, a 50 μL reaction mix was prepared using 25 μL Q5 High-Fidelity 2X Master Mix (New England Biolabs, Inc., Ipswich, MA, USA), 1 μL of each primer, 1 μL of template DNA, and nuclease-free water to adjust the volume. After PCR, DNA concentrations of amplicons (~1500 bp) suitable for purification, were measured. Amplicon sizes were confirmed on agarose gel and purified with AMPure XP nucleic acid purification beads. Each sample was labeled with a separate barcode by following the steps of Native Barcoding Expansion 1-96 (EXP-NBD104) followed by the preparation of sequencing libraries using the Ligation Sequencing Kit (SQK-LSK109) protocols (Oxford Nanopore Technologies Limited, Oxford, UK). Samples were prepared for repairing the DNA ends and adapter ligation, purified with AMPure, and adapter ligation was carried out using the T4 Quick ligase. After further purification, quantification, and flow-cell activation, samples were pooled, subjected to AMPure clean-up, and loaded to R9.4.1 Flow Cell on a MinION Mk1B device to initiate the sequencing (Oxford Nanopore Technologies Limited, Oxford, UK). The DNA sequences were converted to FASTA format for downstream analysis. In order to target the full-length 16S amplicons (~1500 bp), raw sequences were quality-filtered using NanoFilt, and sequences with a q-score less than 7 and length below 1000 bp or above 1800 bp were removed. Taxonomic classification was executed using QIIME 2 followed by mapping the sequences against 16S rDNA reference database SILVA v138. Alpha diversity (Shannon entropy, Chao1 richness, Faith’s Phylogenetic Diversity, and Simpson’s index), and phylum- and genus-based composition analyses were conducted using QIIME 2.

### 2.9. Statistical Analysis

Data were analyzed using a computer-based statistical software package (SPSS version 22.0; IBM Corp. Inc., Armonk, NY, USA). Data were subjected to normality test followed by normalization of non-normalized traits using logarithmic or square root transformation. General linear model procedures were applied to assess the effect of age at weaning and NDF levels in calf starter on growth, serum metabolites, and rumen fermentation characteristics. The differences among the means were assumed significant at 95% probability (*p* < 0.05). In case of significant differences, Tukey’s test was applied as post hoc test to differentiate the significantly different means. Interactions were removed and main effects were considered in case of non-significant interactions. Results were presented as mean ± standard error of the mean (pooled). Following statistical model equation was used to analyze the data:

(1) $$Y_{ijk}\;=\;\mu \;+\;\alpha_{i}\;+\;\beta_{j}\;+\;{\left(\alpha \beta \right)}_{ij}\;+\;e_{ijk}$$

where

*Y_ijk_* = Observed value of the ith level of *α*, *j*th level of *β*, and the *k*th repetition;
*α_i_* = Degree of ith level of *α*;
*β_j_* = Degree of jth level of *β*;
(*αβ*)*_ij_* = Interaction between ith level of *α* and *j*th level of *β*; and
*e_ijk_* = Random error of *Y_ijk_*.

## 3. Results

### 3.1. Growth and Feed Intake

The growth and feed intake of calves subjected to dietary treatments and weaning time are presented in Table 2. The NDF levels of calf starter had no effect on weight gain, final body weight, starter intake, hay intake, and NDF intake of calves. By the end of the experiment, both dietary treatments achieved a similar body weight (14% NDF 108.76 kg vs. 24% NDF 103.99 kg). However, the weaning time significantly affected the weight gain and starter intake at some timepoints. Although overall weight gain was not different in early- and late-weaned calves, late-weaned calves had greater weight gain than their early counterparts (*p* = 0.050) and vice versa (*p* = 0.004). Besides these, starter intake was greater in early-weaned calves (*p* = 0.004) after weaning than late-weaned calves. Early weaning tended to increase the overall starter intake in calves compared to late weaning (123 kg vs. 103 kg, respectively). Final body weight and hay intake of calves were not affected by weaning time. NDF intake was greater in calves fed 24% NDF starter than those fed 14% NDF starter in the pre-weaning (*p* = 0.027), post-weaning (*p* < 0.001), and throughout the experiment (*p* < 0.001). Besides these, calf starter was the main contributor of NDF substrate instead of wheat hay evident from the significant difference in NDF intake contributed by calf starter, being greater in 24% NDF starter receiving calves than their 14% counterparts (*p* < 0.001). Weaning time had no effect on NDF in the pre-weaning phase, overall duration of study, and NDF contribution from calf starter, and wheat hay. However, early-weaned calves had greater NDF intake compared to late-weaned calves (*p* = 0.007).

### 3.2. Rumen Fermentation Characteristics

Rumen pH and NH_3_-N have been shown in Table 3. Rumen pH remained within the physiological range across the groups except for calves receiving 14% NDF starter in the immediate post-weaning period. After weaning, there was a tendency of decreasing rumen pH in 14% NDF starter in comparison with 24% NDF starter whereas, numerically lower pH at all timepoints was seen in calves fed starter with 14% NDF compared to those receiving starter with 24% NDF.

Rumen NH_3_-N concentrations were stable across the treatments at all timepoints. A decreasing trend in rumen NH_3_-N was seen in calves with the progression of age irrespective of treatment.

Molar concentrations of ruminal total VFAs and the proportions of individual VFAs are shown in Table 4. Rumen VFAs before weaning were not affected across the groups. After weaning, a significant interaction for rumen acetic acid was seen between starter NDF levels and weaning time (*p* = 0.013). Rumen isobutyric acid concentration after weaning was greater in calves receiving 24% NDF starter (*p* = 0.001) than those fed 14% NDF starter. At d 90, starter NDF levels and weaning time had no effect on rumen VFAs except isovaleric acid that was greater in early-weaned calves (*p* = 0.044) than late-weaned calves.

### 3.3. Serum Metabolites

Serum metabolites of calves have been depicted in Table 5. All the measured serum metabolites and hepatic markers were within the normal physiological ranges. There was no interaction between starter NDF levels and weaning time at any timepoint. Before weaning, serum TP levels in calves fed starter with 14% NDF were lower (*p* = 0.034) than those fed 24% NDF. Calves receiving 14% NDF starter had lower (*p* = 0.004) serum urea than those receiving 24% NDF starter whereas, early weaned calves had greater (*p* = 0.027) serum urea levels than those weaned later. Serum BHBA levels were greater in calves fed 14% NDF starter (*p* = 0.027) than those fed 25% NDF starter. Early weaned calves had greater serum GGT (*p* = 0.040) and GDH (*p* = 0.027) levels than late-weaned calves. After weaning and at d 90, most serum metabolites were not affected by NDF levels of calf starter and weaning time.

### 3.4. Rumen Microbial Diversity

#### 3.4.1. Alpha Diversity

**Figure 1:**
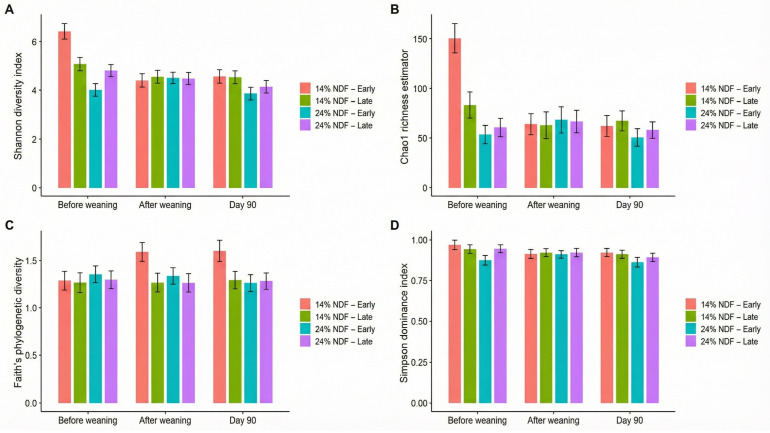
Effect of neutral detergent fiber (NDF) levels in calf starter and weaning time on the alpha diversity of rumen microbes in terms of (**A**) Shannon diversity, (**B**) Chao1 richness, (**C**) Faith phylogenetic diversity, and (**D**) Simpson dominance indices at pre-weaning, post-weaning, and d 90 timepoints of Holstein calves (n = 6). Error bars represent standard error (SE). 14% and 24% represent the NDF levels of calf starter whereas, Early and Late represent the weaning time of calves at d 44 and d 54, respectively.

Alpha diversity of rumen microbiota has been shown in Figure 1. In the pre-weaning phase, starter NDF levels had pronounced effect on the alpha diversity of rumen microbiota of calves weaned early or late. Feeding 14% NDF starter showed distinctly higher (*p* < 0.05) alpha diversity compared to 24% NDF starter, especially in early weaned calves that showed the highest Chao1 richness of 150.7 and Shannon evenness of 6.51 compared to 24% NDF—Early calves (Chao1: 53.0; Shannon: 4.03). Similarly, calves in 14% NDF—Late group had relatively greater Chao1 richness (83.1) (*p* < 0.05) and Shannon evenness (5.12) than those in 24% NDF—Late group (Chao1: 61.0; Shannon: 4.86). A similar trend was noted in Simpson dominance indices of 14% NDF groups vs. 24% NDF groups (0.95–0.98 vs. 0.88–0.95).

During the immediate post-weaning transition, diet-dependent differences among the groups diminished as the alpha diversity indices showed convergence. Groups fed 14% NDF starter exhibited a sharp decline in Chao1 richness (64.0 and 63.0 in early- and late-weaned groups, respectively) whereas, those fed 24% NDF starter showed a numeric increase in Chao1 richness (68.0 and 67.0 in early- and late-weaned calves, respectively). Shannon diversity was seen uniform among the groups ranging narrowly between 4.44 and 4.58 irrespective of the NDF level. Similarly, Simpson index showed similar community dominance among the groups (0.92–0.93).

At the end of experiment, a re-emergence of diet-dependent differences was seen since 14% NDF starter groups conserved stable diversity levels of Chao1 richness (Early weaned: 62.0; Late-weaned: 67.0) and Shannon evenness (Early weaned: 4.58; Late-weaned: 4.57) whereas, 24% NDF starter groups saw a significant decline (*p* < 0.05) in all the alpha diversity indices (Chao1: 50.0 vs. 58.0; Shannon: 3.87 vs. 4.14 in early- and late-weaned calves, respectively).

#### 3.4.2. Phylum Composition

**Figure 2:**
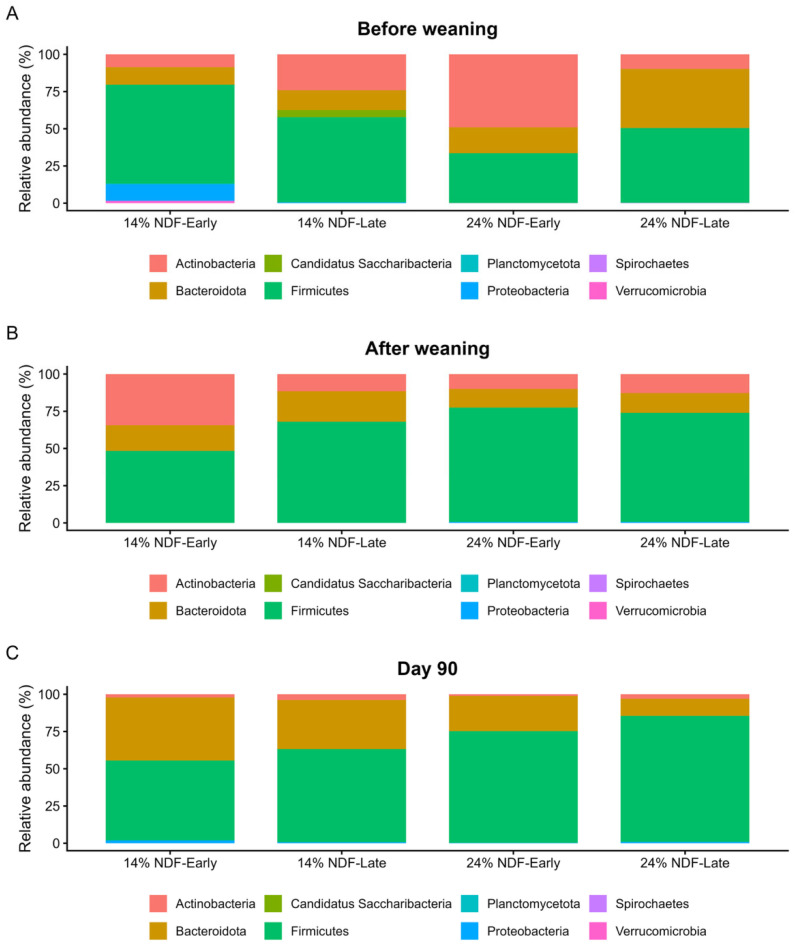
Effect of neutral detergent fiber levels in calf starter and weaning time on the phylum composition of rumen microbes at (**A**) pre-weaning, (**B**) post-weaning, and (**C**) d 90 timepoints of Holstein calves (n = 6). A total of 14% and 24% represent the NDF levels of calf starter, whereas early and late represent the weaning time of calves at d 44 and d 54, respectively.

Relative abundance of major bacterial phyla in the rumen of groups fed different NDF levels in starter and subjected to different weaning times are shown in Figure 2. In the pre-weaning period, rumen microbial community structure was dominated by *Firmicutes*, *Bacteroidota*, and *Actinobacteria*. The 14% NDF—early group was characterized by the highest relative abundance of *Firmicutes* (66.5%) along with a unique spike in *Proteobacteria* (11.1%) which was negligible in other groups. However, microbial community in 14% NDF—late group was co-dominated by *Firmicutes* (57.1%) and *Actinobacteria* (24.1%). In contrast, rumen microbial community in 24% NDF—early group was primarily characterized by *Actinobacteria* (49%) with lowest relative abundance of *Firmicutes* (33.4%) compared to other groups. *Firmicutes* (50.3%) and *Bacteroidota* (39.7%) were dominant in the rumen microbial ecology of 24% NDF—late group.

In the immediate post-weaning period, the groups underwent transition to solid feed-driven divergence. The 14% NDF groups (early- and late-weaned) had a less uniform transition compared to their 24% NDF counterparts. The 14% NDF—early group had a very high relative abundance of *Actinobacteria* (34.4%) and the lowest *Firmicutes* (48.4%) abundance. In contrast, 14% NDF—late group had relatively greater abundance of *Firmicutes* (67.9%) and highest *Bacteroidota* (20.5%). Calves fed 24% NDF starter (weaned early and late) had a microbial community strongly dominated by *Firmicutes* (76.8% and 73.3%, respectively).

At the end of experiment (d 90), a dichotomy of starter NDF levels was seen in the microbial community structure. Calves fed 14% NDF starter (early- and late-weaned) had *Firmicutes* (53.6% and 62.4%, respectively) and *Bacteroidota* (42.4% and 33%, respectively) dominant microbial community. In contrast, microbial community of 24% NDF groups (early and late) was dominated by *Firmicutes* (75% and 84.6%, respectively). Interestingly, relative abundance of *Actinobacteria* had declined to minor levels (<4%).

#### 3.4.3. Genus Composition

**Figure 3:**
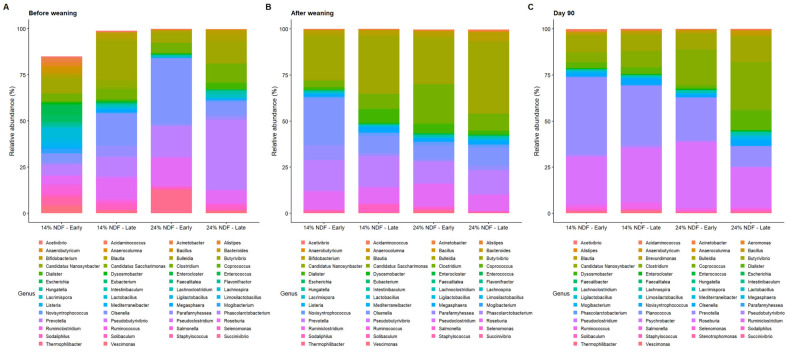
Effect of neutral detergent fiber levels in calf starter and weaning time on the genus composition of rumen microbes at (**A**) pre-weaning, (**B**) post-weaning, and (**C**) d 90 timepoints of Holstein calves (n = 6). 14% and 24% represent the NDF levels of calf starter, whereas early and late represent the weaning time of calves at d 44 and d 54, respectively.

Genera composition in rumen of calves has been shown in Figure 3. In the pre-weaning period, relative abundance of *Prevotella* was highest in 24% NDF—late group (38.1%) followed by 24% NDF—early (16.5%), 14% NDF—late (10.5%), and lowest in 14% NDF—early group (5.5%). *Olsenella* was exceptionally abundant in 24% NDF—early group (35.6%) followed by 14% NDF—late group (17.2%) whereas, it was lower in other groups. *Thermophilibacter* exhibited a spike in 24% NDF—early group (12.3%) while it was negligible in other groups (*p* < 0.05). *Clostridium* was greater in 24% NDF—late group (10.2%) compared to other groups.

In the post-weaning period, relative abundance of *Butyrivibrio* was greater in late-weaned calves fed 14% and 24% NDF starter (31.2% and 38.6%, respectively) than 14% NDF—early group (*p* < 0.05). *Olsenella* was greater in 14% NDF—early group (25.4%) compared to other groups (*p* < 0.05). *Clostridium* was greater in 24% NDF—early group in comparison with other groups (*p* < 0.05). Relative abundance of *Prevotella* and *Roseburia* was similar among the groups.

At the end of experiment (d 90), relative abundance of *Prevotella* was greater in 14% NDF starter groups compared to 24% NDF starter groups (*p* < 0.05). Groups fed 24% NDF starter had *Clostridium* as a major component of microbial community structure than those fed 14% NDF starter (*p* < 0.05). Relative abundance of *Roseburia* was greater in 24% NDF—early group and 14% NDF—late group than 24% NDF—late group (*p* < 0.05). Genera like *Olsenella*, *Thermophilibacter*, and *Parafannyhessea* declined to negligible levels in all groups.

## 4. Discussion

### 4.1. Growth and Feed Intake

It is noteworthy that two different diets not only differed based on different NDF levels but also in their starch content (37% starch in 14% NDF starter vs. 27% starch in 24% NDF starter) and major source of starch (corn in 14% NDF starter vs. barley in 24% NDF starter) implying that the study actually behaved in terms of starch and NDF as major energy sources in starter. In the present study, starter NDF levels had no pronounced effect on final body weight, weight gain, starter intake, and hay intake of calves. Consistent with these findings, a previous study reported that calves fed starter with different NDF levels (18.2% vs. 26.7%) do not differ in live weight [18]. Similar findings were reported in response to the ground and coarse forms of starter and inclusion of forage [19]. In contrast, the study reported that increasing starter NDF levels from 12.85% to 26.99% does not affect the weight gain while further increasing NDF levels to 34% reduced the weight gain in the pre-weaning period, whereas higher starter NDF levels (26.99% and 34%) reduced the weight gain in the post-weaning period [20]. In addition, low and high starter NDF levels (12.85% and 34%) decreased feed intake in claves in pre-weaning period, whereas higher starter NDF levels (26.99% and 34%) lowered the feed intake in calves in the post-weaning period. It has been reported that starchy concentrate starter diets improve the growth performance of pre-weaned calves although the performance of calves is dependent on the starch source too [13,21] reported that using corn-based starter diets improve weight gain, feed intake, and hay intake compared to those based on oats, barley, or wheat [13]. Similarly, replacement of corn with sucrose, molasses, or soybean hulls resulted in reduction in pre-weaning body weight gain of calves except oats [22]. In this study, inclusion of hay had no effect on the growth performance of calves fed different NDF levels. In the same manner, other studies reported no effect of forage supplementation on growth of calves [18,23,24,25,26]. In contrast, several studies have shown that provision of fibrous diets (hay or straw) in results in increased gut fill contributing to reduced intake of energy-rich starter feed [21,26,27] thus affecting the growth. Khan et al. [6] argued that probable gut fill due to forage supplementation in calves may restrict the starter intake; however, a gradual weaning allows a steady commencement of physical and metabolic rumen development in the immediate pre-weaning period in which calves still receive milk that promotes smooth transition from liquid to solid feeds. Our findings suggest that forage supplementation had no effect on starter intake of calves despite the differences in NDF contents of starter and forage supplementation, whereas similar body weights often reflect gut fill instead of tissue mass in fiber fed calves. Interestingly, calves fed 14% NDF starter had relatively greater hay intake than those fed 24% NDF starter suggesting that overall fiber intake was balanced by hay intake. Growth and feed intake, in our study, were likely driven by chemostatic feedback explained in hepatic oxidation theory instead of gut fill. It appears that, despite the differences in starter feeds, equivalence in growth performance between 14% and 24% NDF starter groups might be attributable to two distinct metabolic pathways explained by hepatic oxidation theory of Allen et al. [28] and Allen [29]. The 14% NDF group seemed to have relied primarily on starch fermentation yielding high propionate (NEFA) that acts as a primary secretagogue for insulin that drives amino acid uptake and protein accretion in skeletal muscle as well as triggered the vagus nerve signaling for satiety. On the contrary, 24% NDF group likely compensated via rumen muscularization, passage kinetics (increased passage rate of motile and stronger muscular rumen), and fermentation of fibrous feeds to produce acetate and butyrate that triggered the hepatic signal at the same level of feed intake. A muscular motile rumen might have increased the digesta passage rare by processing more nutrients effectively matching the 14% NDF group resulting in similar growth between the dietary NDF groups.

Extending the milk feeding period in calves (late weaning) improved the weight gain and feed intake in the pre-weaning period followed by a distinct reversal in the post-weaning period in which early weaned calves had greater weight gain and starter intake. Earlier studies have reported similar findings in response to early weaning or prolonged milk feeding in calves [4,6,11,13,30]. In contrast, studies have reported that late weaning in calves results in higher starter intake and greater weight gain compared to early weaning suggesting that delayed weaning in calves not only provides larger body mass that helps buffer the weaning stress for but also enhances smoother transition to adult hepatic metabolism [5,31]. The findings of our study can be explained by the milk intake. Prolonged milk feeding as in late-weaned calves is a key driver of pre-weaning gains by consistently suppressing the pre-weaning starter intake rendering the transition to solid feed metabolically disturbing [30]. Similarly, Khan et al. [13] reported that reducing early milk in life promotes the solid feed intake during the weaning transition and post-weaning resulting in better feed efficiency and post-weaning body weight gain compared to prolonged milk feed feeding (late-weaned calves). Promoting early solid feed intake in calves promotes the growth by boosting the physical rumen development that is essential to sustain high growth rates on milk withdrawal [6]. In addition, promoting early solid feed intake leads to smoother post-weaning transitions by preventing severe hunger and distress as opposed to late weaning [4]. This has been further explained by the early development of starch- and fiber-degrading microbiota in early-weaned calves that helps in starter digestion and better growth in the post-weaning phase [11]. Therefore, the growth performance was better in late-weaned calves in the pre-weaning stage whereas, early-weaned calves grew better in the post-weaning phase in our study.

### 4.2. Rumen Fermentation Characteristics

Measurement of rumen pH, NH_3_-N, and VFAs provided useful insights into the metabolic environment of rumen driven by 14% and 24% NDF starter diets. Rumen pH and NH_3_-N were similar in calves fed 14% or 24% NDF starter although the pH was numerically lower in calves fed 14% NDF starter. While total VFA concentrations were similar, both diets provided energy from fermentable carbohydrates resulting in predictably numerical shifts according to the major carbohydrate source in starter diets. As expected, 14% NDF starter (relatively higher starch) numerically favored the propionate production as opposed to 24% NDF starter (relatively higher fiber) sustained numerically greater concentrations of acetate and butyrate. Stability in ruminal NH_3_-N and iso-acids indicates a degree of synchronization of energy and protein. Our findings are consistent with those of Ren et al. [20] who reported that increased starter fiber levels had no effect on rumen pH and rumen VFA of calves at 70 days of age. A previous study reported that rumen pH remains unaffected in response to partial replacement of concentrate with forage [32]. Furthermore, previous studies have suggested that physically effective fiber in calf diets elevates the rumen pH and supports the greater acetate to propionate ratio [6,24]. All the values for rumen VFA concentrations in our study were within the ranges reported in previous studies [19,24,32,33]. In practice, starch-rich diets tend to reduce the pH by increasing the total VFA concentration in rumen due to increased amylolytic activity, whereas fibrous diets increase the pH due to sustained fibrolytic fermentation which was seen in calves fed 14% NDF starter in the immediate post-weaning timepoint in terms of greater acid load in rumen, risking subacute acidosis. Our study showed that pH did not result in acidosis, whereas NH_3_-N concentrations were similar and sufficient to support optimal microbial protein synthesis. No differences in ruminal pH, NH_3_-N, and VFA despite the differences in diets might be attributable to ad libitum provision of hay to the calves or earlier establishment of rumen microbiota.

Metabolic transition of calves from a pseudo-monogastric to a functioning ruminant is fundamentally dictated by the weaning time. Ruminal pH, NH_3_-N, and VFAs were similar in early- and late-weaned calves except for pronounced numerical differences in total VFAs or individual VFA at some timepoints. Van Ackeren et al. [34] and Rahimi et al. [35] reported similar findings in calves subjected to early or conventional weaning. These findings suggest that weaning time did not pose any threat of acidosis along with sufficient provision of NH_3_-N for microbial protein synthesis. While a compensatory surge in solid feed intake in the post-weaning period of early-weaned calves, it did not alter the fermentation characteristics of rumen indicating the rapid stabilizing ability of rumen regardless of the weaning timeline. It further shows that rumen fermentation is primarily driven by substrate instead of age.

### 4.3. Serum Metabolites

Serum metabolites provide a direct physiological explanation regarding the efficiency of fermentation end-products absorption and metabolism in the calves. In this study, serum metabolites of calves fed 14% and 24% NDF starter were largely dependent on the developmental phase. In the pre-weaning phase, calves receiving 24% NDF starter showed greater levels of serum TP, urea, and BHBA than their 14% NDF counterparts. This confirms that 24% NDF starter stimulated an earlier physical development of rumen wall indicated by the elevated BHBA levels, a biomarker of ruminal ketogenesis and epithelial maturation. Interestingly, these differences disappeared in the post-weaning phase and through 90 days of age, indicating that both starter diets finally supported identical and healthy nutritional status in terms of protein and energy in the post-weaning period. These findings coincide with those of Ren et al. [20] who reported that high starter NDF levels (27% and 34%) result in higher BHBA levels at d 70 compared to low starter NDF levels (13% and 20%) whereas, these differences disappeared at d 112. Increased BHBA levels in 24% NDF starter group in the pre-weaning stage might be attributed to fibrolytic fermentation yielding butyrate that is absorbed and metabolized into BHBA before entering the bloodstream [1,36]. In our study, serum BHBA levels increased with the increasing calf age consistent with the previous studies [37,38]. In contrast, a previous study has reported that serum BHBA levels tend to decline as the calves grow [6]. A simultaneous increase in serum TP and urea indicates efficient protein degradation and cycling of nitrogen for growth. This corresponds well with the better growth of calves fed 24% NDF starter compared to 14% NDF starter. In the post-weaning phase until day 90, 14% NDF group were likely forced to ferment their high-starch starter bringing their glucose, BHBA, and urea levels to match 24% NDF group.

Transition from a liquid milk diet to solid feed is a stressful period that requires reprogramming of metabolic apparatus in calves. There are very limited number of studies describing the effect of weaning age on serum metabolites of calves. In the present study, early weaning initiated a rapid metabolic shift in the pre-weaning phase in terms of higher concentrations of urea and liver enzymes such as GGT and GDH in comparison with late-weaned calves. In addition, a strong tendency of higher serum glucose was seen in early-weaned calves in the pre-weaning phase. Interestingly, nearly all the metabolic differences disappeared in the post-weaning phase and through d 90 indicating that early- and late-weaned calves managed to achieve metabolic homeostasis immediately after weaning. Similar results were reported by Ferronato et al. [39] in early-weaned calves; however, liver enzymes remained unaffected irrespective of weaning age. Increase in urea along with GGT and GDH in early-weaned calves in the pre-weaning phase are indicative of increasing protein metabolism, gluconeogenesis, and adaptation of liver to sudden influx of rumen fermentation products since GDH enzyme removes amino group from amino acids in order to transfer to urea cycle while remaining carbon skeleton is funneled into gluconeogenesis [40]. This also explains the relatively greater serum glucose in early-weaned calves in the pre-weaning phase. Similarly, GGT regulates the amino acid transport across cellular membranes and manages to overcome oxidative stress in high metabolic output [41].

### 4.4. Rumen Microbial Diversity

In the pre-weaning period, rumen microbial diversity was highly variable with prominent ecological divergences based on diet. Calves fed 14% NDF starter had much broader microbial richness and diversity primarily led by *Firmicutes* (57.1–66.5%) followed by *Bacteroidota* (11.78–13.34%), *Actinobacteria* (8.59–24.1%), and *Proteobacteria* (0.62–11.07%) with no clear majority of genera. In contrast, 24% NDF starter restricted the richness and diversity with phylum *Actinobacteria* (49.03%) driven by genus *Olsenella* (35.56%) in 24% NDF—Early group as opposed to its late-weaned counterpart that exhibited a spike in *Bacteroidota* (39.7%) dominated by *Prevotella* (38.13%). These findings indicate the 14% NDF starter created a harsh rumen environment indicated by high richness and diversity indices possibly due to milk feeding higher starch in 14% NDF starter diets. However, higher abundance of *Olsenella* and *Prevotella* in early- and late-weaned calves fed 24% NDF starter reflect the transition from milk to starter in these groups. These findings are consistent with those of Jami et al. [42] who demonstrated that pre-weaning rumen microbiome is unstable and highly prone to extreme opportunistic blooms before complete transition to solid feed that establishes distinct fermentation pathways. Similarly, Rey et al. [43] described that pre-weaning rumen microbial community is highly unstable but represents key functional microbes for degradation followed by solid feed-driven maturation. They further described that *Prevotella* becomes dominant as solid feed intake increases whereas earlier appearing genera decreased or disappeared. Our findings are likely attributable to undeveloped rumen that acts as an unstable fermentation since liquid feed was primarily processed in the abomasum whereas, rumen lacked the consistent supply of fermentable solid carbohydrates to develop a strict microbial hierarchy. Besides these, 14% NDF starter, being rich in starch, ensured the supply of a readily available energy that possibly supported the development of a diverse, however, opportunistic community. Conversely, 24% NDF starter restricted the microbial diversity due to the inability of immature rumen to degrade the structural carbohydrates leading to the overgrowth of lactic-acid producing *Olsenella* or early starch-scavenger *Prevotella*. This might have suppressed the alpha diversity in the rumen of calves fed high-fiber starter in the pre-weaning phase.

In the post-weaning phase, extreme variability of pre-weaning phase vanished as the weaning process equalized the microbial ecology of rumen. Alpha diversity converged (unification) across all the groups to a stable uniform state with a profound taxonomic shift towards *Firmicutes* dominance at phylum-level, driven by a universal surge in *Butyrivibrio* which was the dominant genus among the groups. A previous study demonstrated that weaning in calves stabilizes the alpha diversity and selects for adaptable cross-feeding *Firmicutes* that manage the successful transition from liquid to solid feed [11]. In addition, Flint et al. [44] *Butyrivibrio* initiate a quick response to the sudden influx of plant origin polysaccharides which explains the convergence of alpha diversity and universal selection towards this genus due to the removal of milk (weaning). The convergence in alpha diversity in the post-weaning phase to *Firmicutes* phylum and *Butyrivibrio* is likely the response of rumen microbial communities to the weaning. As the diet was forced to shift from liquid to solid starter in this study, active fermentation that drops rumen pH might have forced anaerobic environment that contributed to the alleviation of chaotic pre-weaning transient. Moreover, weaning mandated the rumen ecosystem to supply energy and rapid development of rumen epithelium to absorb that enforced the universal selection for *Butyrivibrio*, a genus capable of fermentation of substrates like hemicellulose and starch into butyrate. Butyrate is responsible for maturation and development of rumen wall. Therefore, it is thought that the microbial diversity and ecology in the post-weaning phase in this study was primarily driven by weaning.

At the end of experiment (day 90), a diet-driven polarization emerged based entirely on dietary NDF levels whereas, the effect of weaning time had completely vanished. Although alpha diversity was stable, 14% NDF starter groups had greater richness than their 24% NDF counterparts. The diet-based bifurcation was evident from the phylum and genus composition of groups. Calved fed 24% NDF starter had dominant *Firmicutes* (74.9–84.6%) and suppressed *Bacteroidota* (11.3–23.6%) as opposed to 14% NDF starter that maintained a robust *Firmicutes* (53.6–62.4%) dominated by *Roseburia* (25.9–29.6%) in addition to *Bacteroidota* (32.9–41.6%) dominated by genus *Prevotella* (32.3–41.7%). In contrast, 24% NDF starter groups suppressed the genus *Prevotella* (10.9–23.1%) and selected for *Roseburia* (21.5–36.1%) and *Clostridium* (19.2–25.6%). These findings are consistent with those of Henderson et al. [45] who reported that diet composition dictated by ratio of structural to non-structural carbohydrates determines the *Firmicutes* to *Bacteroidota* ratio in dairy cows. Our findings are also consistent with those of Zened et al. [46] who reported that high dietary fiber enriches the cellulolytic *Clostridia* and butyrate-producing genera like *Roseburia*. Similarly, consistent with our study, Stevenson and Weimer [47] reported that high concentrate diet results in the selection of *Prevotella*. In contrast, Petri et al. [48] revealed that core rumen microbiome is very resilient to dietary changes in terms of NDF as well as higher forage ratios that maintains a stable *Prevotella* dominance in heifers as opposed to our study that showed a massive suppression in the relative abundance of *Prevotella* in 24% NDF starter. In the present study, rumen microbial diversity seemed to have adapted to the respective diets by the end of experiment whereas, the weaning effect from post-weaning phase had completely vanished. Calves receiving 14% NDF starter rich in readily fermentable starch had massive abundance of *Prevotella* (starch degraders) that help the starch degradation by secreting amylase enzymes showing that the rumen of these calves specialized into amylolytic metabolism. In contrast, those receiving 24% NDF starter high in fiber content specialized into fibrolytic rumen metabolism evident by fiber degrading genera like *Clostridia* and *Roseburia*. *Clostridia* physically degrade the plant cell walls releasing intermediate by-products that were possibly further converted into butyrate by *Roseburia*. These findings indicate that microbial ecology of rumen in calves at d 90 was dictated by the chemical composition of the diet rather than the weaning time.

In the present study, serum metabolites demonstrated a functional linkage between the diet, resulting ruminal microbial diversity, and systemic metabolism in calves. Transient increase in serum TP, urea, and liver enzymes like GGT and GDH in early-weaned calves corresponded directly with establishment of amylolytic microbial populations, especially the dominance of *Prevotella* in 14% NDF groups. *Prevotella* genus is known for rapid starch fermentation as well as protein degradation. This likely resulted in the surge in ruminal propionate thereby forcing the immature liver towards upregulation of gluconeogenesis and urea cycle that manifested as temporary spikes in circulating urea and liver enzymes as hepatic stress indicators. Conversely, elevated relative abundance of *Clostridium* in 24% NDF groups in the pre-weaning phase perfectly mirrored the elevated serum BHBA levels since *Clostridium* are fiber degraders that feed *Roseburia* and *Butyrivibrio* that primarily produce butyrate. It implies that 24% NDF starter groups degraded the higher starter fiber to produce butyrate which was absorbed and converted into BHBA that entered the bloodstream thus the corresponding spike in serum BHBA in 24% NDF group was seen. This linkage between the microbial diversity and systemic metabolism in calves started converging in the immediate post-weaning phase that was established by the end of this study evident by the similar serum metabolites at these timepoints.

## 5. Conclusions

Under the conditions of the present study, it is concluded that dietary NDF levels did not affect growth and feed intake in calves whereas, early- and late-weaned calves adapted to achieve the comparable long-term growth. Besides these, rumen fermentation characteristics remained largely unaffected by dietary NDF levels and weaning time. However, blood metabolites demonstrated changes driven by NDF levels and weaning time in the pre-weaning phase indicating a transition towards rumen development and maturation driven both by the diet and the weaning time. The study further demonstrated that rumen microbial diversity in the pre-weaning phase (liquid feeding) is highly rich and unstable driven by diet although solid feed intake is minimal during this phase. Nonetheless, rumen microbial diversity and ecology became stable and driven solely by the weaning in the immediate post-weaning phase evident by the convergence of alpha diversity as well as phylum and genera composition among the groups. Finally, rumen microbial diversity and ecology in late post-weaning phase (day 90) presented a clear bifurcation based on the composition of diet that drove the specialization of rumen microbes towards the low (starch rich) and high NDF (fiber rich) diets. Based on the physiological and ecological adaptations, it is concluded that calves can be subjected to early weaning with a moderately high-fiber (24% NDF) calf starter.

## Figures and Tables

**Table 1:** Ingredient and nutrient composition (%) of calf starters and wheat hay.

Item	14% NDF	24% NDF	Wheat Hay
Maize	48	10	—
Barley	5	30	—
Oats	—	8	—
Sunflower meal, 36% CP	2.4	20	—
Soybean meal, 46% CP	25	5.4	—
Wheat bran	11	14	—
Maize gluten	—	3	—
By-pass fat	—	1	—
Molasses	5	5	—
Salt	1	1	—
Limestone	1	1	—
Dicalcium phosphate	1.5	1.5	—
Vitamin-mineral premix	0.1	0.1	—
Nutrient composition (%, dry matter basis)			
Dry matter	87.89	89.83	92.75
Crude protein	19.00	19.00	8.12
Crude fiber	4.20	8.0	25.91
Ether extract	2.89	3.20	2.63
Neutral detergent fiber	14.02	24.86	32.77
Acid detergent fiber	5.45	11.00	18.15
Starch	37.06	27.39	—
Crude ash	6.76	6.96	10.29
Calcium	0.91	0.91	—
Phosphorus	0.72	0.75	—
Metabolizable energy, kcal/kg	2750	2720	—

**Table 2:** Effect of neutral detergent fiber levels in calf starter and weaning time on the growth and feed intake of Holstein calves (n = 6).

Item ^1^	Initial Body Weight (kg)	Weight Gain (kg)			Final Body Weight (kg)	Starter Intake (kg)			Hay Intake (kg)	NDF Intake (kg)				
		Pre-Weaning	Post-Weaning	Total		Pre-Weaning	Post-Weaning	Total		Pre-Weaning	Post-Weaning	Total	Starter NDF	Hay NDF
NDF														
14%	43.250	26.497	38.199	64.696	108.76	26.182	86.051	112.234	9.317	3.671	15.118	18.788	15.735	3.053
24%	42.833	23.196	36.685	59.881	103.99	23.717	90.704	114.422	7.748	5.896	25.088	30.984	28.445	2.539
Weaning														
Early	43.417	21.530	42.416	63.946	108.37	21.562	101.622	123.184	8.976	4.230	23.049	27.278	24.337	2.941
Late	42.667	28.163	32.468	60.631	104.38	28.338	75.133	103.471	8.089	5.337	17.157	22.494	19.844	2.651
SEM	1.620	2.252	2.184	3.859	4.88	3.773	5.735	8.883	1.269	0.660	1.395	1.945	1.642	0.416
*p*-value														
NDF	0.857	0.312	0.629	0.388	0.497	0.649	0.573	0.863	0.392	0.027	<0.001	<0.001	<0.001	0.392
Weaning	0.746	0.050	0.004	0.550	0.569	0.219	0.004	0.132	0.627	0.249	0.007	0.097	0.067	0.627
NDF × Weaning	0.801	0.520	0.412	0.402	0.480	0.477	0.317	0.343	0.122	0.684	0.056	0.126	0.154	0.122

^1^ NDF = neutral detergent fiber; SEM = standard error of the mean.

**Table 3:** Effect of neutral detergent fiber levels in calf starter and weaning time on the rumen pH and ammonia nitrogen concentration of Holstein calves (n = 6).

Item ^1^	pH				Ammonia Nitrogen (mg/dL)		
	Pre-Weaning	Post-Weaning	Day 90		Pre-Weaning	Post-Weaning	Day 90
NDF							
14%	5.65	5.47	6.47		10.77	9.88	5.79
24%	5.96	5.69	6.67		10.20	9.53	4.31
Weaning							
Early	5.80	5.58	6.69		11.22	10.16	4.37
Late	5.82	5.58	6.45		9.75	9.25	5.72
SEM	0.12	0.08	0.12		0.87	0.69	0.55
*p*-value							
NDF	0.780	0.090	0.232		0.644	0.928	0.069
Weaning	0.890	0.984	0.158		0.247	0.814	0.095
NDF × Weaning	0.628	0.371	0.483		0.321	0.156	0.838

^1^ NDF = neutral detergent fiber; SEM = standard error of the mean.

**Table 4:** Effect of neutral detergent fiber levels in calf starter and weaning time on the rumen total volatile fatty acids (mmol/L) and proportion of individual volatile fatty acids (%) of Holstein calves (n = 6).

Item ^1^	Pre-Weaning ^2^								Post-Weaning								Day 90						
	TVFA	AA	PA	IBA	BA	IVA	VA		TVFA	AA	PA	IBA	BA	IVA	VA		TVFA	AA	PA	IBA	BA	IVA	VA
NDF																							
14%	55.94	52.07	33.44	0.28	7.69	0.27	6.26		63.43	54.08	31.25	0.16	8.03	0.26	6.23		43.27	53.70	32.81	1.19	7.17	1.03	4.10
24%	44.97	55.18	29.82	0.39	7.20	0.32	7.09		52.14	51.28	32.97	0.42	7.95	0.41	6.98		39.28	55.03	31.67	1.02	7.22	1.31	3.76
Weaning																							
Early	56.76	53.04	32.40	0.26	7.19	0.23	6.89		57.51	52.63	31.30	0.30	8.00	0.43	7.35		36.34	55.05	31.45	1.30	6.98	1.51	3.71
Late	44.15	54.22	30.86	0.41	7.70	0.35	6.46		58.06	52.73	32.92	0.28	7.98	0.23	5.86		46.21	53.68	33.03	0.91	7.40	0.83	4.16
SEM	5.83	1.59	1.38	0.08	0.60	0.08	1.47		7.79	1.60	1.49	0.05	0.56	0.16	2.14		4.18	1.24	1.02	0.35	0.37	0.22	0.44
*p*-value																							
NDF	0.198	0.209	0.096	0.319	0.596	0.693	0.709		0.318	0.258	0.454	0.001	0.930	0.537	0.818		0.507	0.458	0.439	0.742	0.920	0.395	0.590
Weaning	0.142	0.626	0.465	0.198	0.579	0.309	0.849		0.961	0.967	0.477	0.829	0.983	0.423	0.646		0.110	0.441	0.289	0.443	0.430	0.044	0.483
NDF × Weaning	0.255	0.731	0.779	0.232	0.872	0.509	0.958		0.250	0.013	0.690	0.509	0.134	0.193	0.224		0.669	0.887	0.986	0.381	0.240	0.260	0.206

^1^ NDF = neutral detergent fiber; SEM = standard error of the mean. ^2^ TVFA = Total VFA; AA = Acetic acid; PA = Propionic acid; IBA = Isobutyric acid; BA = Butyric acid; IVA = Isovaleric acid; VA = Valeric acid.

**Table 5:** Effect of neutral detergent fiber levels in calf starter and weaning time on serum metabolites of Holstein calves ^1^ (n = 6).

Item	Pre-Weaning								Post-Weaning								Day 90						
	TP	Urea	TC	Glu	BHBA	GGT	GDH		TP	Urea	TC	Glu	BHBA	GGT	GDH		TP	Urea	TC	Glu	BHBA	GGT	GDH
NDF																							
14%	4.47	4.18	93.5	94.98	0.06	7.67	50.43		5.61	7.82	75.33	89.21	0.13	6.92	85.25		6.76	13.09	39.16	107.18	0.22	6.92	86.19
24%	6.28	7.63	132.9	125.93	0.09	11.92	74.80		4.99	9.09	78.97	72.93	0.12	4.71	61.93		6.16	11.98	50.93	105.04	0.22	7.17	137.77
Weaning																							
Early	6.11	7.16	134.6	129.13	0.08	13.58	94.15		4.66	7.69	67.59	70.08	0.12	5.83	73.83		6.22	13.15	38.26	96.98	0.20	6.17	109.30
Late	4.65	4.66	91.9	91.78	0.07	6.00	31.08		5.94	9.22	86.70	92.06	0.13	5.79	73.34		6.69	11.92	51.83	115.24	0.24	7.92	114.65
SEM	0.53	0.69	13.8	12.06	0.01	2.28	17.43		0.57	0.93	11.58	7.46	0.02	0.76	23.59		0.64	2.06	4.81	8.29	0.02	0.85	26.40
*p*-value																							
NDF	0.034	0.004	0.073	0.104	0.027	0.230	0.364		0.474	0.375	0.836	0.163	0.612	0.070	0.518		0.521	0.708	0.099	0.857	0.977	0.838	0.182
Weaning	0.080	0.027	0.054	0.054	0.189	0.040	0.027		0.152	0.288	0.286	0.065	0.660	0.970	0.989		0.611	0.678	0.060	0.135	0.203	0.163	0.887
NDF × Weaning	0.072	0.568	0.736	0.252	0.059	0.230	0.788		0.651	0.562	0.981	0.673	0.565	0.340	0.697		0.506	0.843	0.425	0.725	0.666	0.381	0.818

^1^ TP = Total protein (g/dL); TC = Total cholesterol (mg/dL); Glu = Glucose (mg/dL); BHBA = Beta-hydroxy butyric acid (mmol/L); GGT = Gamma-glutamyltransferase (U/L); GDH = Glutamate dehydrogenase (U/L).

## Data Availability

The data presented in this study are available on request from the corresponding author.

## Abbreviations

The following abbreviations are used in this manuscript:

VFAs	Volatile fatty acids
NDF	Neutral detergent fiber
ANDFom	Ash-free neutral detergent fiber after amylase treatment
ADFom	Ash-free acid detergent fiber
TP	Total protein
GLU	Glucose
BUN	Blood urea nitrogen
CHOL	Cholesterol
BHBA	Beta-hydroxybutyric acid
GDH	Glutamate dehydrogenase
GGT	Gamma-glutamyl transferase
NH_3_-N	Ammonia nitrogen
